# Antihyperglycemic Effect of *Orthosiphon Stamineus* Benth Leaves Extract and Its Bioassay-Guided Fractions

**DOI:** 10.3390/molecules16053787

**Published:** 2011-05-04

**Authors:** Elsnoussi Ali Hussin Mohamed, Ali Jimale Mohamed, Mohd. Zaini Asmawi, Amirin Sadikun, Omar Saad Ebrika, Mun Fei Yam

**Affiliations:** 1 School of Pharmaceutical Sciences, Universiti Sains Malaysia, 11800, Penang, Malaysia; E-Mails: gelyac2@gmail.com (A.J.M.); amzaini@usm.my (M.Z.A.); amirin@usm.my (A.S.); edri7@yahoo.com (O.S.E.); 2 Faculty of Medicine and Health Sciences, Universiti Putra Malaysia, 43400, Selangor, Malaysia; E-Mail: yammunfei@yahoo.com

**Keywords:** *Orthosiphon stamineus*, antihyperglycemic, hypoglycemic

## Abstract

Preliminary investigations were carried out to evaluate the antidiabetic effects of the leaves of *O. stamineus* extracted serially with solvents of increasing polarity (petroleum ether, chloroform, methanol and water); bioassay-guided purification of plant extracts using the subcutaneous glucose tolerance test (SbGTT) was also carried out. Only the chloroform extract, given at 1 g/kg body weight (b.w.), significantly reduced (*P* < 0.05) the blood glucose level of rats loaded subcutaneously with 150 mg/kg (b.w.) glucose. The active chloroform extract of *O. stamineus* was separated into five fractions using a dry flash column chromatography method. Out of the five fractions tested, only chloroform fraction 2 (Cƒ2), at the dose of 1 g/kg (b.w.) significantly inhibited (*P* < 0.05) blood glucose levels in SbGTT. Active Cƒ2 was split into two sub-fractions Cƒ2-A and Cƒ2-B, using a dry flash column chromatography method. The activities Cƒ2-A and Cƒ2-B were investigated using SbGTT, and the active sub-fraction was then further studied for anti-diabetic effects in a streptozotocin-induced diabetic rat model. The results clearly indicate that Cƒ2-B fraction exhibited a blood glucose lowering effect in fasted treated normal rats after glucose-loading of 150 mg/kg (b.w.). In the acute streptozotocin-induced diabetic rat model, Cƒ2-B did not exhibit a hypoglycemic effect on blood glucose levels up to 7 hours after treatment. Thus, it appears that Cƒ2-B functions similarly to metformin, which has no hypoglycemic effect but demonstrates an antihyperglycemic effect only in normogycemic models. The effect of Cƒ2-B may have no direct stimulatory effects on insulin secretion or on blood glucose levels in diabetic animal models. Verification of the active compound(s) within the active fraction (Cƒ2-B) indicated the presence of terpenoids and, flavonoids, including sinensitin.

## 1. Introduction 

*Orthosiphon stamineus* Benth [syn: *O. aristatus*(B1) Miq., *O. grandiflorus* Bold., *O. spicatus* (Thumb) Bak.; Lamiaceae] is known locally in Malaysia as Misai Kucing. *O. stamineus* is also found in other locations such as Thailand, Indonesia and Europe. In these places, Misai Kucing is also known as Yaa Nuat Maeo, Rau Meo or Cay Bac (Thailand); Kumis Kucing or Remujung (Indonesia); Moustaches de Chat (France); or Java Tea (Europe) [1]. To date, *Orthosiphon stamineus* Benth [1] is a popular traditional folk medicine extensively used in Southeast Asia for the treatment of a wide range of diseases. It is used in Indonesia for rheumatism, diabetes, hypertension, tonsillitis, epilepsy, menstrual disorders, gonorrhea, syphilis, renal calculus and gallstones [2]; in Vietnam for urinary lithiasis, edema, eruptive fever, influenza, hepatitis, jaundice and biliary lithiasis [3]; and in Myanmar to alleviate diabetes and urinary tract and renal diseases [4]. 

Due to its popularity and demonstrated effectiveness, phytochemical studies [5,6,7] and pharmacological studies [8,9,10] of this plant have been conducted since the 1930s, and highly-oxygenated isopimarane-type diterpenes, orthosiphols A-E, were reported, in addition monoterpenes, triterpenes, saponins, flavonoids, hexoses, organic acids, rosmarinic acid, chromene and myo-inositol. *O. stamineus* has been reported to possess hypoglycemic and antihyperglycemic activity [11], with an aqueous extract producing a significant hypoglycemic effect in normal and STZ-induced diabetic rats. This present study aimed to elucidate any antihyperglycemic or hypoglycemic effects of the plant, which was extracted serially with solvents in normal and diabetic rats. This method of extraction helps to separate the content of the plant according to the polarity of the solvent. The blood glucose profile and dynamics of extract-treated rats were then studied using the subcutaneous glucose tolerance test (S-bGTT). 

In theory, these tests should able to screen hypoglycemic and antihyperglycemic agents. Hypoglycemic agents are those that are capable of reducing blood glucose levels to below fasting levels, whereas antihyperglycemic agents lowers blood glucose levels, but not beyond the fasting level. Glibenclamide is a hypoglycemic agent while metformin is an antihyperglycemic or euglycemic agent. The approach used in this study was intended to prevent any false-negative or false-positive findings in screening antidiabetic plants and also, to continue screening with SbGTT-guided fractionation and identify the chemical group(s) of the active fraction.

## 2. Results 

### 2.1. Hypoglycemic test 

**Figure 1:**
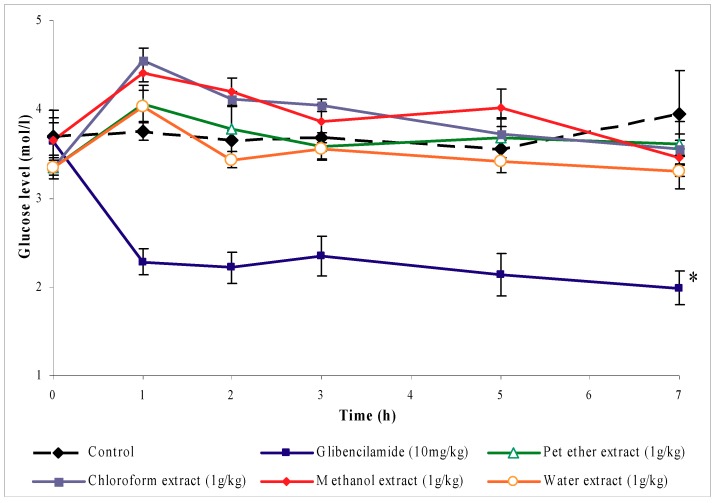
The effect of oral administration of petroleum ether, chloroform, methanol, and water extracts of *O. stamineus* leaves 1 g/kg (b.w.) on blood glucose levels of normal rats. The values are given as mean ± s.e.m (n = 6). * indicates a significant difference (*P* < 0.05) between the control and glibenclamide treated groups. (The treatments were given orally 1 h before time 0).

Figure 1 shows that oral treatment with petroleum ether, chloroform, methanol and water extracts of *O. stamineus,* 1 g/kg (b.w.), did not significantly alter the blood glucose levels in normal rats, as compared to the control group, during the 7 hour time course of the experiment, whereas the blood glucose levels in glibenclamide-treated rats were significantly reduced (*P* < 0.01) from the first hour until the 7^th^ hour after treatment. 

### 2.2. Subcutaneous Glucose Tolerance Test (SbGTT) guided fractionation

#### 2.2.1. Effect of *O. stameineuse* extracts 

**Figure 2:**
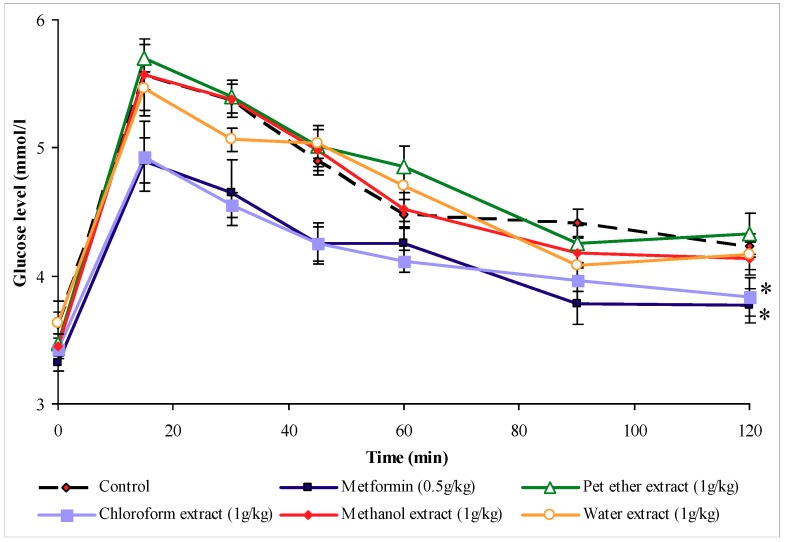
The effect of oral administration of petroleum ether, chloroform, methanol, and water extracts of *O. stamineus* leaves 1 g/kg (b.w.) on blood glucose levels of normal rats loaded with glucose 150 mg/kg (b.w.) subcutaneously. The values are given as mean ± s.e.m (n = 6). * indicates a significant difference (*P* < 0.05) between the control group and metformin and chloroform extract treated groups. (The treatments were given orally 1 h before time 0 and glucose was loaded at time 0).

Figure 2 shows that treatment with petroleum ether, methanol, and water extracts did not significantly inhibit the rise in blood glucose levels of glucose-loaded rats. However, increases in blood glucose levels of chloroform extract- and metformin-treated rats were significantly inhibited (*P* < 0.05) after glucose loading, as compared to the control group.

#### 2.2.2. Effects of different fractions of chloroform extract 

**Figure 3:**
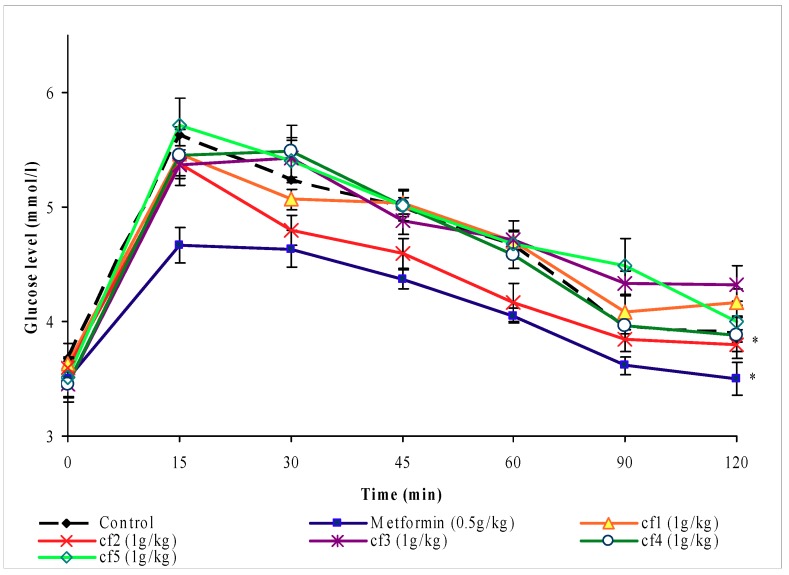
The effect of oral administration of chloroform fractions Cƒ1, Cƒ2, Cƒ3, Cƒ4 and Cƒ5 1 g/kg (b.w.) on blood glucose levels of normal rats loaded with glucose 150 mg/kg (b.w.) subcutaneously. The values are given as mean ± s.e.m (n = 6). * indicates a significant difference (*P* < 0.05) between the control group and metformin and fraction Cƒ2 treated groups. (The treatments were given orally 1 h before time 0 and glucose was loaded at time 0).

There were no significant differences (*P* > 0.05), compared to the control group, in the blood glucose levels of normal rats treated with 250 or 500 mg/kg of fractions Cƒ1, Cƒ2, Cƒ3, Cƒ4 or Cƒ5 from the chloroform extract after subcutaneous glucose loading (data not shown). However, when the dose was increased to 1 g/kg (b.w.), the blood glucose levels of the glucose-loaded rats treated with fraction Cƒ2 and metformin were significantly lower (*P* < 0.05) than those of control group (Figure 3).

#### 2.2.3. Effects of the sub-fractions of fraction 2 of chloroform extract

**Figure 4:**
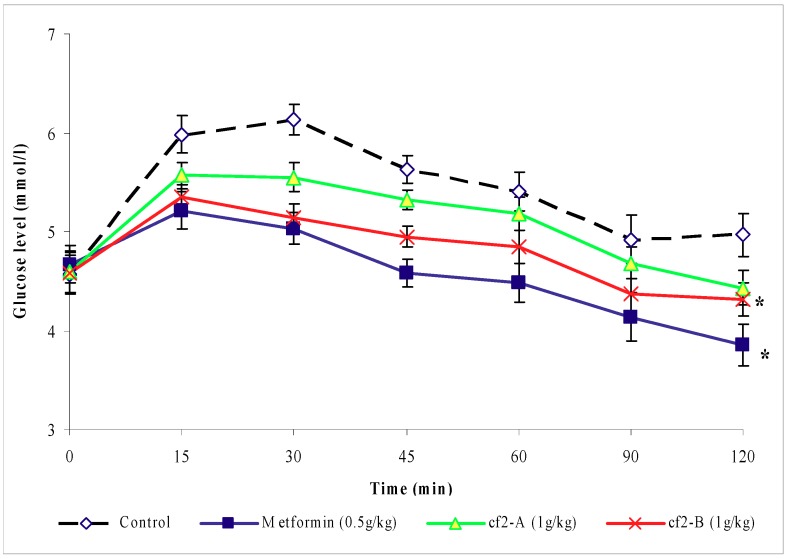
The effect of oral administration of sub-fractions Cƒ2-A and Cƒ2-B 1g/kg (b.w.) on blood glucose levels of normal rats loaded with glucose 150 mg/kg (b.w.) subcutaneously. The values are given as mean ± s.e.m (n = 6). * indicates a significant difference (*P* < 0.05) between the control group and metformin and sub-fraction Cƒ2-B treated groups. (The treatments were given orally 1 h before time 0 and glucose was loaded at time 0).

The changes in blood glucose levels of glucose-loaded rats treated with sub-fractions Cƒ2-A and B are shown in Figure 4 The blood glucose levels of rats treated with sub-fraction Cƒ2-B were significantly lower (*P* < 0.05) than those of the control group. There was no significant difference (*P* > 0.05) in blood glucose levels of Cƒ2-A treated rats compared to the control group. However, the rise in the blood glucose levels of the metformin- treated glucose-loaded rats were significantly lower (*P* < 0.05) than that of the control group.

### 2.3. Effects of Cƒ2-B on serum insulin levels 

**Figure 5:**
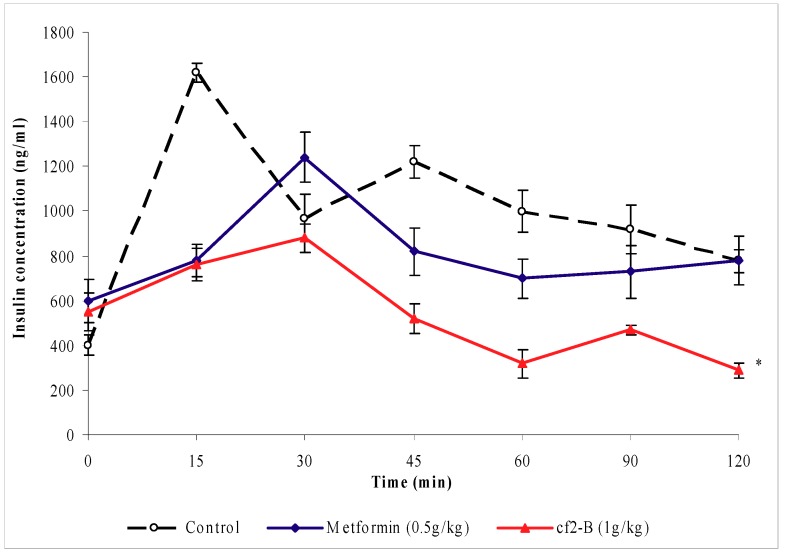
The effect of oral administration of sub-fraction Cƒ2-B 1 g/kg (b.w.) on plasma insulin levels of normal rats loaded with glucose 150 mg/kg (b.w.) subcutaneously. The values are given as mean ± s.e.m (n = 6). * indicates a significant difference (*P* < 0.05) between the control group and metformin and sub-fraction Cƒ2-B treated groups.

Figure 5 shows that the insulin levels of control rats began to rise immediately after glucose loading and reached its peak 15 minutes later. After this period, the plasma insulin levels of normal rats began to decrease gradually. However, the insulin levels of both metformin- and Cƒ2-B- treated rats were significantly reduced (*P* < 0.05) and were lower than those of the control rats. 

### 2.4. Effects of Cƒ2-B on streptozotocine-induced diabetic rats

**Figure 6:**
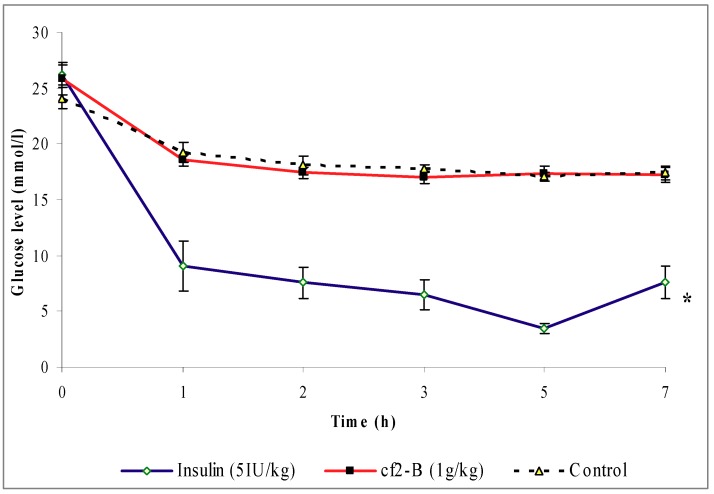
The effect of oral administration of sub-fraction Cƒ2-B 1 g/kg (b.w.) on blood glucose levels of Streptozotocin-induced diabetic rats. The values are given as mean ± s.e.m (n = 6). * indicates a significant difference (*P* < 0.05) between the control and insulin treated groups.

Oral treatment with 1 g/kg (b.w.) Cƒ2-B failed to reduce the blood glucose concentration to below control levels (Figure 6), and there was no significant difference (*P* < 0.05) in the blood glucose levels of Cƒ2-B treated and control group rats over the time course of the study. However, the blood glucose levels of the rats treated with insulin, 5 IU/kg (b.w.) were significantly reduced (*P* < 0.05).

### 2.5. Purification of chloroform extract

Five fractions (Cƒ1: 20.26%, Cƒ2: 30.11%, Cƒ3: 12.50%, Cƒ4: 11.58% and Cƒ5: 14.30%) were obtained from the fractionation of the chloroform extract of *O. stamineus* leaves using dry flash-column chromatography. Further separation of the active fraction gave two sub-fractions (Cƒ2-A: 25.6% and Cƒ2-B: 36.0%).

### 2.6. Phytochemical investigation of Cƒ2-B

**Figure 7:**
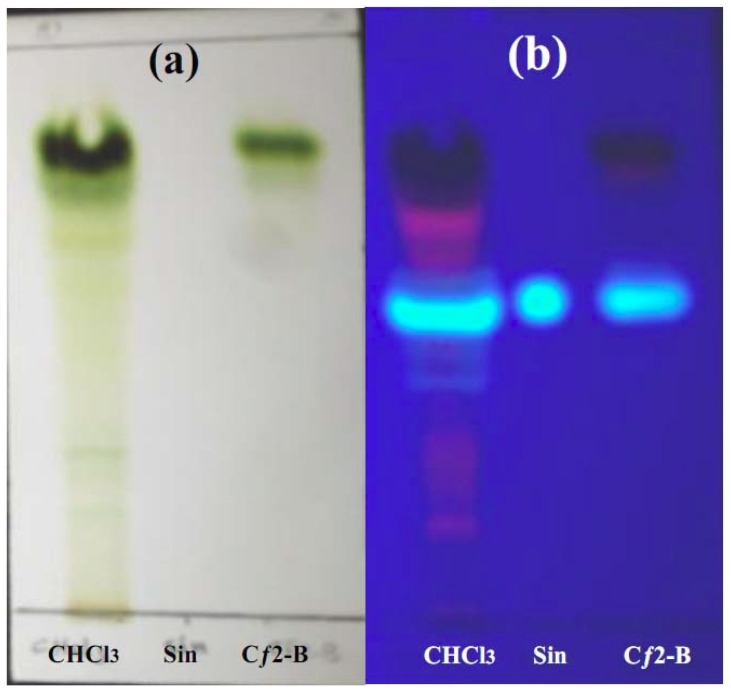
TLC profile of sub-fraction (Cƒ2-B), chloroform extract (CHCl3) and sinensitin (Sin) after being developed with ethyl acetate: chloroform (7.5:2.5) as mobile phase and sprayed with natural product (NP/PEG) reagent under (a) visible light and (b) UV 365 nm.

**Figure 8:**
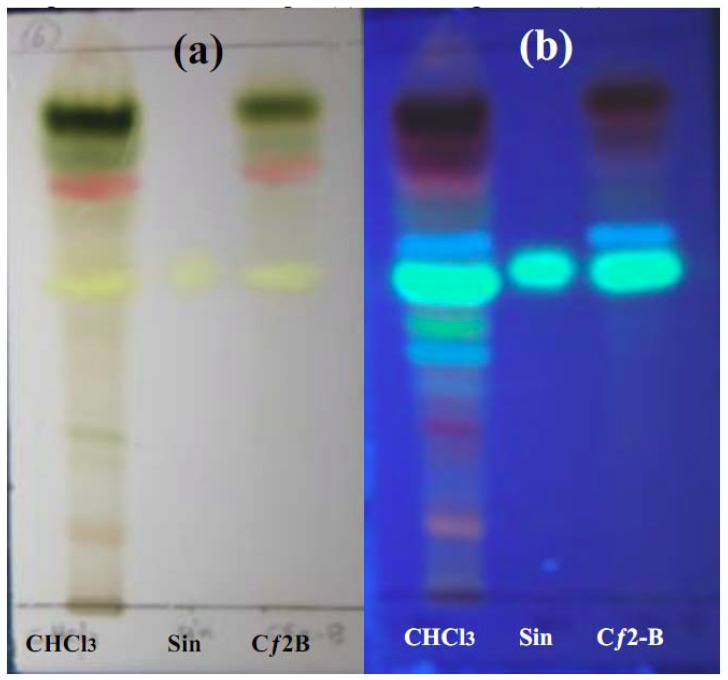
TLC profile of sub-fraction (Cƒ2-B), chloroform extract (CHCl3) and sinensitin (Sin) after being developed with ethyl acetate: chloroform (7.5:2.5) as mobile phase and sprayed with Antimony trichloride reagent under visible light (a) visible light and (b) UV 365 nm.

Phytochemical screening of Cƒ2-B indicated the presence of terpenoids and flavonoids. One of the flavonoids identified was sinensitin, while alkaloids and coumarins were absent (Figure 7 and Figure 8).

## 3. Discussion 

The hypoglycemic activity of leaf extracts of the *O. stamineus* plant was first evaluated with the hypoglycemic test. A novel result obtained in this study indicates that the oral administration of leaves extracts, at 1 g/kg (b.w.), to normal fasting rats had no hypoglycemic activity, as indicated by their failure to reduce the glucose levels of normal rats below the fasting level concentrations. Glibenclamide (an oral hypoglycemic agent), on the other hand, lowered the blood glucose level to below the fasting level and caused hypoglycemia. Glibenclamide acts by stimulating insulin release from pancreatic β-cells [12]. Another action that may contribute to the hypoglycemic effect of glibenclamide is suppression of glucagons release [13].

The second evaluation of the antihyperglycemic activity of *O. stamineus* extracts utilized the subcutaneous glucose tolerance test. The results obtained from the primary screening of the leaves extracts of *O. stamineus* showed that petroleum ether, methanol and water extracts, dosed at 1 g/kg (b.w.), failed to inhibit the rise in blood glucose levels of glucose-loaded fasting rats. Under the same conditions however, the chloroform extract yielded a significant decrease in the hyperglycemia induced by subcutaneous glucose loading in the normal rats. This suggests that the chloroform extract may have antihyperglycemic activity by improving the glucose tolerance in the treated animals. This finding is consistent with the previous investigation of *O. stamineus* extracts [11]. Mariam *et al.*, observed that the aqueous extract of this plant, given 1 g/kg (b.w.), inhibited the rise of blood glucose levels of normal rats loaded with glucose. In the present study, SbGTT shows the blood glucose levels of the chloroform extract-treated group in a dynamic perspective, with the glucose tolerance curve reaching the fasting level within two hours. From this result, it appears that the chloroform extract functions as an antihyperglycemic agent. It was decided to further fractionate the chloroform extract of *O. stamineus* and continue screening with activity-guided fractionation to identify the active compound(s) responsible for this activity. The separation produced five fractions: Cƒ1, Cƒ2, Cƒ3, Cƒ4 and Cƒ5. It was found that, at the dose of 1 g/kg (b.w.), only fraction Cƒ2 screened positive using the subcutaneous glucose tolerance test. This result showed that the antihyperglycemic activities of the chloroform extract are due to Cƒ2. Moreover, using continuous screening with activity-guided fractionation, the active fraction (Cƒ2) was re-fractionated to finally yield two sub-fractions: Cƒ2-A and Cƒ2-B. The results clearly indicate that when the duration of blood glucose lowering induced by equal doses of 1g/kg (b.w.) Cƒ2-A and Cƒ2-B are compared, the latter appears to be more potent. Cƒ2-B exhibited a blood glucose lowering effect in fasting, treated rats, when compared to appropriate controls, after glucose loading of 150 mg/kg (b.w.). Metformin also inhibited the rise of blood glucose levels. These findings show the similarity between the effects of Cƒ2-B and metformin as antihyperglycemic agents. Metformin efficacy requires the presence of insulin and involves several therapeutic effects [14]. Some of these effects are mediated via increased insulin action, while some are not directly insulin-dependent. Metformin reduces gluconeogenesis by potentiating the effect of insulin, reducing hepatic extraction of certain substrates (e.g. lactate) and opposing the effect of glucagon [15]. In addition, metformin also reduces the overall rate of glycogenolysis, decreases the activity of hepatic glucose-6-phosphatase and enhances insulin-stimulated glucose uptake into skeletal muscle. This effect has been attributed in part to increased movement of insulin-sensitive glucose transporters into the cell membrane [16,17]. Other mechanism involved in the blood glucose-lowering effects of metformin include an insulin-independent suppression of fatty acid oxidation and a reduction of blood triglyceride levels. These effects reduce the energy supply for gluconeogenesis and subsequently regulate the glucose-fatty acid cycle [17]. The active compound of Cƒ2-B may function similarly to metformin, a biguanide compound.

The streptozotocin-induced diabetic rat model used in this study replicates the treatment responces of Type I Diabetes Mellitus. In this model, only subcutaneous insulin is able to reduce the blood glucose level. Screening of fraction Cƒ2-B of *O. stamineus* at the dose of 1 g/kg (b.w.) shows an antihyperglycemic effect in SbGTT in normal rats. However, in the diabetic model used, there was no antihyperglycemic effect of Cƒ2-B on blood glucose levels up to 7 hours after treatment, as compared to the control group. Insulin was able to significantly reduce the blood glucose levels at 1, 2, 3, and 5 hours post-treatment, as compared to control group. These results suggest that Cƒ2-B functions similarly to metformin, which does not have a hypoglycemic effect but does have an antihyperglycemic effect in normal animal models. A preliminary phytochemical analysis of Cƒ2-B, by TLC and using specific reagents, was achieved according to previously published methods [18]. The results showed that the extract contained; terpenoids and flavonoids and that one of the flavonoids identified was sinensitin, which was present in the crude and in sub-fraction Cƒ2-B. The presence of flavonoids such as eupatorin, rutin, and 5-hydroxy-6, 7, 3′,4′-tetramethoxyflavone in *O. stamineus* are well established [11,19,20]. Moreover, Merck and Mustaq *et al*. [21,22] have reported that flavonoids and terpenoids possessed hepatoprotective and hypoglycemic activities. In addition, Oliver-Bever [23] listed glycosides, flavonoids, tannins, organic sulfur compounds, catechol and alkaloids as active ingredients in hypoglycemic plants. Furthermore, Babu *et al*. [24], during their investigation on the antidiabetic activity of *Cassia klenii* leaves using isolation of an active fraction, reported that the antihyperglycemic activity of the *Cassia klenii* plant was found predominantly in the chloroform fraction of the alcohol extract, which contained terpenoids, coumarins and saponins. Acting separately or synergistically, sinensitin and other compounds present in the Cƒ2-B fraction isolated during this study could be responsible for the antihyperglycemic effect of Cƒ2-B. 

## 4. Experimental 

### 4.1. Reagents

Chemicals and reagents used in the present studies were of analytical grade and purchased from Sigma Chemical Co. (St. Louis, MO, USA) or Merck (Darmstadt, Germany), Metformin was obtained as 500 mg tabs supplied by UPHA^®^_._ Human insulin, 100 IU/mL, was purchased from Novo Nordisk (Copenhagen, Denmark). Thin layer chromatography plates were obtained from Merck (TLC plate Art-5554, Merck). Blood glucose levels were determined using the Accu-Cehek Advantage II clinical glucose meter (Roche Diagnostics Co., Corporation 9115 Hague Read Indianapolis, IN 46256, USA). Insulin concentration in the plasma samples was assayed by enzyme-linked immunoassay (ELISA) using the Rat Insulin ELISA Kit (Crystal Chem, Corporate Headquarters 1536 Brook Drive, Suite A Downers Grove, IL, USA). 

### 4.2. Animals

Normoglycemic female Sprague-Dawley rats weighing 200–250 g were used in this study and were obtained from the animal house of the School of Pharmaceutical Sciences, Universiti Sains Malaysia, Penang. The animals were kept at 25–30 °C and 45–55% relative humidity and were acclimatized with free access to food (Golden feed, Delhi, India) and water *ad libitum* for 1 week under a 12 h light, 12 h dark cycle. All animals were carefully monitored, and all experimental work with animals was carried out after obtaining approval from the Institutional Animal Ethical Committee. For experimental purposes, animals were kept fasting overnight but had free access to water.

### 4.3. Plant material and preparation of extracts 

The plant *O. stamineus* Benth was collected from Kepala Batas, Pulau Pinang Malaysia (June 2004). It was identified by En. Adenan Jaafar, School of Biological Sciences, Universiti Sains Malaysia, and a voucher specimen (10810) was deposited at the Herbarium of School of Biological Sciences, Universiti Sains Malaysia. The plant was washed and then dried at room temperature for two days. The dried leaves were then ground in an electric grinder to a coarse powder and weighed. The dried powder materials (300 g) were immediately extracted by the serial extraction method in a Soxhlet, using petroleum ether (5L) followed by chloroform (5L) and then methanol (5L) (8 h each solvent at 40–65 °C) and finally by water (5L) using the maceration method in 60 °C in water bath for three days (yield: petroleum ether 8.5%, chloroform 5.6%, methanol 12.0% and water 5.2%). The extracts were concentrated using a Büchi-RE121 evaporator (Büchi Laboratorium-Technik AG, BUCHI Labortechnik AG Meierseggstrasse 40 Postfach CH-9230 Flawil 1, Switzerland) equipped with a Büchi-B169 vacuum system, and then dried in a Hetovac VR-1 (HETO Lab Equipment, Gydevang 17-19, 3450, ALLERØD, Denmark) freeze dryer. The lyophilized extracts were then kept in desiccators in a refrigerator (0–4 °C) prior to use in our experiments.

### 4.4. Hypoglycemic test 

Six groups of normal female Sprague-Dawley rats (n = 6) of 200–250 g body weight were fasted overnight. The first group was treated orally with 5 mL/kg (b.w.) saline and served as a negative control. The second group was given 10 mg/kg (b.w.) glibenclamide as a positive control. Groups three to six were treated orally with 1 g/kg (b.w.) of petroleum ether, chloroform, methanol or water extracts of *O. stamineus*, respectively. Blood samples were drawn from the rats’ tail veins before treatment and at 1, 2, 3, 5 and 7 hours after oral treatment. Blood glucose levels were determined using the Accu-Chek Advantage II clinical glucose meter. 

### 4.5. Subcutaneous glucose tolerance test (SbGTT)

The subcutaneous glucose tolerance test (S.bGTT) was conducted on normal female S.D rats weighing 200–250 g (b.w.) according to the method of Zhang and Tan *et al*. [25]. Six groups of animals (n = 6) were fasted over night and treated orally with 500 mg/kg (b.w.) metformin as a positive control, 5 mL/kg (b.w.) saline as a negative control or one of the plant material extracts (petroleum, chloroform, methanol and water extracts) at 1 g/kg (b.w.). One hour later, all the animals were given a glucose load of 150 mg/kg (b.w.) subcutaneously. Blood samples were drawn from the rats’ tail before glucose injection and at 15, 30, 45, 60, 90 and 120 minutes after the administration of glucose. Blood glucose levels were measured using the Accu-Chek Advantage II clinical glucose meter.

### 4.6. Fractionation of chloroform extract

The active chloroform extract (160 g) was fractionated using dry flash column chromatography. Ten grams of chloroform extract was pre-adsorbed onto the adsorbent (silica gel-1:2) by first dissolving the extract in 300 mL of chloroform. Then, silica gel (20 g, 7730, Merck) was added to the solution and mixed well. The solvent was then evaporated using a rotary evaporator to produce a dried sample. The mixture was placed onto the top of the column and packed evenly by applying suction. The column was first pre-eluted under vacuum with 2 × 300 mL of 100% petroleum ether. The column was then eluted with the following solvents: 2 × 200 mL pet-ether-chloroform (7:3), 2 × 200 mL pet-ether-chloroform (1:1), pet-ether-chloroform (3:7), 2 × 200 mL chloroform (100%), 2 × 200 mL chloroform-methanol (7:3), 2 × 200 mL chloroform-methanol (1:1), 2 × 200 mL chloroform-methanol (3:7) and 2 × 200 mL methanol (100%). The fractions obtained from different solvent systems were collected in different pre-labeled conical flasks (150 mL each). The fractions then examined via thin layer chromatography (TLC) (using mobile phase = ethyl acetate:chloroform (7:4)) and those giving the same profiles were combined, affording five fractions labeled as Cƒ1, Cƒ2, Cƒ3, Cƒ4 and Cƒ5 (yield 20.28%, 11%, 15.50%, 11.58% and 14.30% of chloroform extract, respectively).

The active chloroform fraction 2 (40.5 g) was sub-fractioned using similar flash column chromatography methods as those described above with different elution solvents: 3 × 50 mL 100% petroleum ether, 3 × 50 mL 65% petroleum ether-chloroform, 4 × 50 mL 35% petroleum ether-chloroform, 3 × 50 mL 100% chloroform, 3 × 50 mL 60% of chloroform-methanol, 4 × 50 mL 40% chloroform-methanol and 5 × 50 mL of 100% methanol. All the fractions collected were examined by TLC (using mobile phase = ethyl acetate:petroleum ether (7.5:2.5)), and those giving the same profiles were pooled together affording five fractions labeled as Cƒ2-A and Cƒ2-B (yield 25.6% and 36% of chloroform extract fraction, respectively).

### 4.7. Subcutaneous glucose tolerance test (SbGTT) guided fractionation

The second round of bioactivity screening with subcutaneous glucose tolerance test was performed on chloroform fractions (Cƒ1, Cƒ2, Cƒ3, Cƒ4 and Cƒ5) and sub-fractions (Cƒ2-A and Cƒ2-B) 1 g/kg (b.w.) of fraction 2 of chloroform Extract of *O. stamineus* using the same procedure mentioned above (Section 4.5). 

### 4.8. Determination of serum insulin

The rats were treated as in Section 3.4 with the active sub-fraction Cƒ2-B, but the blood samples collected were used for the insulin assay instead of blood glucose level determination. The amount of blood samples collected from the rat’s tail vein at 0, 15, 30, 45, 60, 90 and 120 minutes after glucose loading was 75 µL each time. These blood samples were collected into hematocrit-capillary tubes (Hirschmann Laborgerate GmbH & Co. KG, Eberstadt, Germany) and centrifuged at 12,000 rpm for 3 minutes. The plasma samples obtained were stored at −20 °C until measured for insulin concentration. The insulin concentration in the plasma samples was assayed by enzyme-linked immunoassay (ELISA) using the Rat Insulin ELISA Kit (Crystal Chem) [26].

### 4.9. Induction of experimental diabetes

Hyperglycemia was induced in rats by a single intraperitoneal (i.p) injection of streptozotocin [STZ, 65 mg/kg (b.w.)] [27]. The STZ was freshly dissolved in citrate buffer (0.01 M, pH 4.5) and kept on ice prior to use; the injection volume was 1 mL/kg. A week after streptozotocin administration, hyperglycemic was confirmed in STZ-treated rats with fasting blood glucose values over 300 mg/dL [28].

### 4.10. Hypoglycemic test in acute streptozotocine-induced diabetic rats

Four groups of diabetic rats (n = 6), each weighing 200–250 g (b.w.), were fasted over-night. The first group was treated orally with 5 mL/kg (b.w.) saline as a control, the second group was treated with 500 mg/kg (b.w.) Cƒ2-B, the third group was treated with 1 g/kg (b.w.) Cƒ2-B and the fourth group was given insulin 5 IU/kg (b.w.) i.p. Blood samples were drawn from rats’ tail vein before treatment (0 hours) and at 1, 2, 3, 5 and 7 hours after treatment. Blood glucose levels were determined using the Accu-Chek Advantage II clinical glucose meter (Roche Diagnostics Co.). 

### 4.11. Phytochemical investigation of active sub-fraction Cƒ2-B of chloroform extract 

Phytochemical tests were carried out for various constituents of chloroform extract (as crude) and sub-fraction (Cƒ2-B) in comparison with the reference compound (sinensitin) using the following chemicals and reagents: Dragendoff’s reagents for alkaloids, natural product reagent for flavonoids, antimony trichloride reagent for terpenoids and sulphuric acid reagent for coumarins [18]. 

### 4.12. Statistical analysis

All data was expressed as mean ± s.e.m. Statistical analysis of data was performed by two-way analysis of variance (ANOVA). The differences between the means were considered significant at the probability level *P* < 0.05. The statistical analysis was done using the computer program SPSS (Release 11.5, SPSS Inc., 2001).

## 5. Conclusions 

In conclusion, Cƒ2-B may have no direct stimulatory effects on insulin secretion, and may produce hypoglycemia through an extra-pancreatic mechanism, probably by increasing peripheral utilization of glucose by the tissues. Moreover, as this study is still in preliminary stages and only focuses on the acute aspects of pharmacology, further comprehensive pharmacological and biochemical investigation should be carried out to elucidate the mechanism(s) of the antidiabetic effect of this plant. Useful studies would include conducting anti-diabetic activity guided-isolation of *O. stamineus* and phytochemically screening the classes of active compound(s).
